# *GALNT1* Expression Is Associated with Angiogenesis and Is a Prognostic Biomarker for Breast Cancer in Adolescents and Young Adults (AYA)

**DOI:** 10.3390/cancers15133489

**Published:** 2023-07-04

**Authors:** Masanori Oshi, Danya Ziazadeh, Rongrong Wu, Kohei Chida, Akimitsu Yamada, Shinya Yamamoto, Kazutaka Narui, Li Yan, Takashi Ishikawa, Itaru Endo, Kazuaki Takabe

**Affiliations:** 1 Department of Surgical Oncology, Roswell Park Comprehensive Cancer Center, Buffalo, NY 14263, USA; masa1101oshi@gmail.com (M.O.); danyazia@buffalo.edu (D.Z.); kohei.chida@roswellpark.org (K.C.); 2 Department of Gastroenterological Surgery, Yokohama City University Graduate School of Medicine, Yokohama 236-0004, Japan; ayamada@yokohama-cu.ac.jp (A.Y.);; 3 Department of Breast Surgery and Oncology, Tokyo Medical University, Tokyo 160-8402, Japan; rwuren@gmail.com (R.W.); tishik55@gmail.com (T.I.); 4 Department of Breast and Thyroid Surgery, Yokohama City University Medical Center, Yokohama 232-0024, Japan; yamashin@yokohama-cu.ac.jp (S.Y.); nr158@yokohama-cu.ac.jp (K.N.); 5 Department of Biostatistics & Bioinformatics, Roswell Park Comprehensive Cancer Center, Buffalo, NY 14263, USA; li.yan@roswellpark.org; 6 Division of Digestive and General Surgery, Niigata University Graduate School of Medical and Dental Sciences, Niigata 951-8520, Japan; 7 Department of Breast Surgery, Fukushima Medical University School of Medicine, Fukushima 960-1295, Japan; 8 Department of Surgery, Jacobs School of Medicine and Biomedical Sciences, State University of New York, Buffalo, NY 14263, USA

**Keywords:** angiogenesis, AYA, breast cancer, *GALNT1*, gene expression, signaling, Tn antigen, tumor microenvironment, survival, biomarker

## Abstract

**Simple Summary:**

Breast cancer (BC) in adolescents and young adults (AYA) has unique biological qualities, but the details of these features remain unclear, and treatment options are comparable to those of other age groups. In this study, we identified a gene that demonstrates a strong association with patient prognosis solely in AYA patients, despite the fact that its expression does not differ by age group. Although this gene, *GALNT1*, has no prognostic significance in whole BC, when examined in AYA BC, high *GALNT1* was significantly associated with worse survival. Furthermore, high *GALNT1* expression was found to be an independent factor for survival among several clinical features of AYA patients. Finally, high *GALNT1* BC is associated with increased EMT, angiogenesis, and protein secretion in AYA patients, but not in the elderly. We report the clinical relevance of the *GALNT1* gene in AYA BC.

**Abstract:**

It is well established that genetic information differs amongst the adolescent and young adult population (AYA) and older patients. Although several studies on genetic information have been conducted, no current prognostic biomarker exists to help differentiate survival outcomes amongst AYA patients. The GALNT family of genes have been associated with several cancer etiologies, such as the Tn antigen and epithelial-mesenchymal transition (EMT); however, the clinical significance of *GALNT1* expression in breast cancer (BC) remains unclear. We investigated the clinical relevance of *GALNT1* expression in BC using two large independent cohorts. We found that, although triple-negative BC (TNBC) had the highest *GALNT1* expression compared to ER-positive/HER2-negative BC, *GALNT1* levels in BC were not associated with clinical aggressiveness, including histological grade, AJCC stage and N-category, and patient survival, consistently in both the METABRIC and GSE96058 cohorts. There was also no biological difference between low- and high-*GALNT1* expression BC, as analyzed by hallmark gene sets via gene set enrichment analysis (GSEA). Further, no significant difference was found in *GALNT1* expression levels among AYAs and older patients. However, high *GALNT1* expression was associated with significantly worse survival in AYA patients, in both cohorts. Furthermore, high *GALNT1* expression was found to be an independent factor among several clinical features, including subtype, histological grade, AJCC T and N-category, in AYA patients. In both cohorts, BC with high *GALNT1* expression demonstrated low levels of CD8^+^ T-cell infiltration, but not other anti-cancerous or pro-cancerous immune cells. Finally, high levels of *GALNT1* BC demonstrated increased EMT, angiogenesis, and protein secretion in the AYA population, but not in older patients. In conclusion, our findings demonstrate that *GALNT1* expression was found to be associated with angiogenesis and EMT, and may have potential as prognostic biomarker, specifically in AYA patients.

## 1. Introduction

“AYA” is a frequently utilized acronym for the adolescent and young adult population, which is commonly defined as patients younger than 40 years old. Approximately 89,000 AYA are diagnosed with thyroid, skin, colorectal, and breast cancer in the United States each year, accounting for about 5% of all cancers diagnosed in the country. Although the incidence of breast cancer increases with age, AYA patients account for 5% of all breast cancer cases in the United States yearly, equivalent to about 12,000 cases per year and 4000–5000 per year in Japan [1,2]. Further, breast cancer remains the leading cause of cancer incidence and mortality among AYA, according to SEER [3]. Breast cancer in AYA patients is known to be clinically aggressive, with larger tumor size, higher likelihood of lymph node metastases, advanced stages, and aggressive subtype, TNBC or HER2-positive [4]. Breast cancer in this population is strongly associated with family history and genetic germline alterations, and also presents unique social challenges, such as fertility preservation [5]. Survival outcomes are often worse in AYA patients compared to older patients [3], and thus require more intensive therapies. Given these differences, there have been multiple studies examining gene expression amongst AYA and older patients. However, there is currently no biomarker that can identify prognosis and survival outcomes in AYA patients.

One of the methods in this biomarker research is investigation of gene expression profile to study the root cause of cancer; however, methods in approaching the analysis of huge amounts of data has been difficult. In recent years, the adaptation of machine learning and artificial intelligence-based approaches have made remarkable breakthroughs in analyzing big data and identifying biomarkers using high-throughput genomic data [6]. Furthermore, several bioinformatical algorithms, such as gene set enrichment analysis [7,8], xCell [9], CIBERSORT [10], TIMER [11], and ESTIMATE [12], helps us better understand a new perspective of tumor biology.

*GALNT1* belongs to a family of polypeptide GalNAc transferases (GALNTs), and is the enzyme that initiates GalNAc-type O-glycosylation by transferring GalNAc to threonine or serine polypeptide residues of various glycoproteins, including the UMC1, Sonic hedgehog protein (Shh), and osteopontin [13,14]. O-glycosylation stabilizes the MUC1 gene, which plays a role in breast carcinogenesis [15]. Additionally, O-glycosylation of Osteopontin—an extracellular glycoprotein implicated in tumor development—advances its phosphorylation, cell-adhesion properties, and cancer progression [16]. Lastly, aberrant GalNAc-type O-glycosylation by *GALNT1* in the endoplasmic reticulum drives accumulation of truncated O-glycans, such as the Tn antigen.

Cancer cells have been demonstrated to express much higher levels of the Tn antigen compared to surrounding non-cancer cells [17,18]. The Tn antigen, well-recognized in its role in cancer progression, has been implicated in stimulating cell adhesion, driving cell migration and invasion, and promoting the formation of metastases [19]. Most notably, it has been found in about 90% of breast cancer [20,21]. It has also been found to play a role in other cancers. The Tn antigen suppresses the tumor immune microenvironment and drives colorectal cancer growth [22], and its expression has been correlated with colorectal cancer metastases and prognosis [23]. A similar correlation has been evidenced in lung cancer [24]. Yet, the role of the Tn antigen remains controversial, with some studies reporting its association with a poorer prognosis amongst breast cancer patients [25], with other studies demonstrating no significant relationship [26].

Although there was a report that high *GALNT1* expression was significantly associated with decreased survival in breast cancer [14], this study used optimal cut-off and the results were not validated. In our current study, we investigated the clinical significance of *GALNT1* expression in 5176 breast cancer patients from the METABRIC (*n* = 1903) and GSE96058 (*n* = 3273) cohorts using multiple computational algorithms.

## 2. Materials and Methods

### 2.1. Data Acquisition of Breast Cancer

A total of 5176 breast cancer patients with clinical information and corresponding mRNA transcriptomic data were extracted from the Molecular Taxonomy of Breast Cancer International Consortium (METABRIC) [27] and GSE96058 [28] cohorts. The clinical features of patients including age, subtype, pathological grade, AJCC stage, topography (T), and lymph node (N) were downloaded. The Roswell Park Institutional Review Board waived approval due to the deidentified nature of the data. Overall survival (OS) was recorded from the time of study enrollment to the day of death due to any cause or last follow-up. Disease-specific survival (DSS) was defined as the time between the day of diagnosis or initiation of treatment for breast cancer and the day of death due to breast cancer.

### 2.2. Biological Function Analysis

To investigate the biological function of *GALNT1* expression, we used a gene set variation analysis (GSEA) algorithm [7] with gene sets from the Molecular Signatures Database (MSigDB) hallmark gene set collection [29], as we reported previously [30,31,32,33,34]. The significantly enriched pathways were analyzed based on a false discovery rate (FDR) < 25%, as recommended by GSEA.

### 2.3. Other Scores

Cytolytic activity score (CYT) was defined as the geometric mean of granzyme A (*GZMA*) and perforin (*PRF1*) expression values in Transcripts Per Milion (TPM), as we previously reported [35,36,37]. An xCell algorithm was used to estimate the fraction of sixty-four immune and stromal cell types in each tumor tissue to evaluate intra-tumor microenvironment composition. These cell fractions were calculated via their online calculator, as we previously reported [38,39,40,41,42].

### 2.4. Statistical Analysis

The threshold of dichotomization of low and high *GALNT1* expression groups was determined by the top one-fourth points within each cohort. The correlations between the expression of *GALNT1* and clinic pathologic features were conducted using the Kruskal–Wallis and Mann–Whitney U tests. Comparisons of several immune infiltrating cells, including CD8^+^ T cells, CD4^+^ T cells, Th1 and Th2 cells, M1 and M2 macrophages, as well as CYT score, between low and high *GALNT1* expression groups were performed with the Mann–Whitney U test. The association between *GALNT1* expression and patient survival, along with other clinicopathological features, was analyzed utilizing univariate and multivariate analysis with Cox regression analysis. All statistical analyses and visualization were performed using R software (ver4.1.0) and Microsoft Excel (ver16), with a *p* value < 0.05 indicating statistical significance.

## 3. Results

### 3.1. GALNT1 Expression Levels Were Not Associated with Clinicopathological Aggressiveness, Patient Outcomes, or Biological Function in the Whole Breast Cancer Cohort

**Figure 1:**
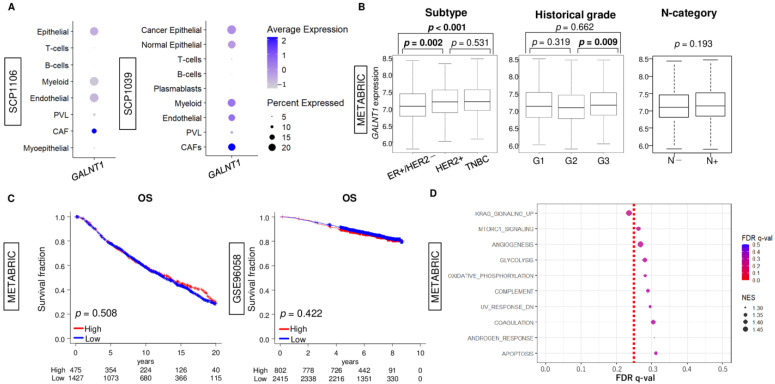
Association of *GALNT1* expression with clinicopathological features, breast cancer patient survival, and biological function. (**A**) Boxplots of *GALNT1* expression by cancer cells, immune cells; T cells, B cells, Myeloid, and stromal cells; epithelial, endothelial, cancer-associated fibroblasts (CAF), plasma blasts, and perivascular-like (PVL) cells, in the SCP1106 and SCP1039 single-cell sequence cohorts. (**B**) Boxplots of *GALNT1* expression by subtype (ER-positive/HER2-negative, HER2-positive, and triple-negative breast cancer (TNBC)), histological grade (G1–G3), and lymph node metastasis (N-negative and N-positive) in METABRIC cohort. Significant difference among groups was calculated by the Kruskal–Wallis and Mann–Whitney U tests. (**C**) Kaplan–Meier plots of overall survival (OS) in breast cancer patients in the METABRIC, and OS in GSE96058 cohorts by low (blue) and high (red) *GALNT1* expression groups. Significant difference of survival between groups was calculated by log-rank test. The top one-fourth was used as a cut-off to divide low and high groups for each cohort. (**D**) Dot plots of the ten gene sets with the lowest FDR q-value in the enrichment analysis between low and high *GALNT1* low and high breast cancer groups using the GSEA algorithm. The top one-fourth was used as a cut-off to divide low and high groups.

Since *GALNT1* generates the Tn antigen that is widely reported to be predominantly expressed in cancer cells [17,18], there is clinical interest in investigating cellular expression levels in the tumor microenvironment (TME) of breast cancer patients, utilizing single-cell sequence cohorts. We found that the cancer cells, along with some other cells such as immune and stromal cells, were the main sources accounting for the observed *GALNT1* expression in bulk tumors (Figure 1A). We investigated *GALNT1* expression in tumors by subtype, Nottingham histological grade, and lymph node metastasis, and enrichment of the hallmark gene sets in the METABRIC and GSE96058 cohorts. We found that *GALNT1* expression was increased in triple-negative breast cancer (TNBC) and HER2-positive subtypes compared to ER-positive/HER2-negative BC, consistently in both cohorts, although this difference was minor. There was no consistent trend in *GALNT1* expression by histological grade although it appeared lower in G2 in the METABRIC cohort (Figure 1B) and higher in G1 in the GSE96058 cohort (Supplementary Figure S1). Furthermore, *GALNT1* expression level was not significantly different by lymph node metastases in the METABRIC cohort, whereas it was in the GSE96058 cohort (Figure 1B and Supplementary Figure S1). Despite significant differences in *GALNT1* expression levels among subtypes, there was no difference in patient overall survival (OS) *GALNT1* expression levels divided by the top quarter (Figure 1C). Further, none of the 50-hallmark gene sets, except for KRAS signaling pathway, enriched to high *GALNT1* expression tumors by gene set enrichment analysis (GSEA) (Figure 1D). These results demonstrated that *GALNT1* expression level was not strongly associated with clinicopathological or biological features in breast cancer as a whole cohort.

### 3.2. High Levels of GALNT1 Expression in Breast Cancer Was Significantly Associated with Decreased Survival in Adolescent and Young Adults (AYA), but Not in Older Patients

**Figure 2:**
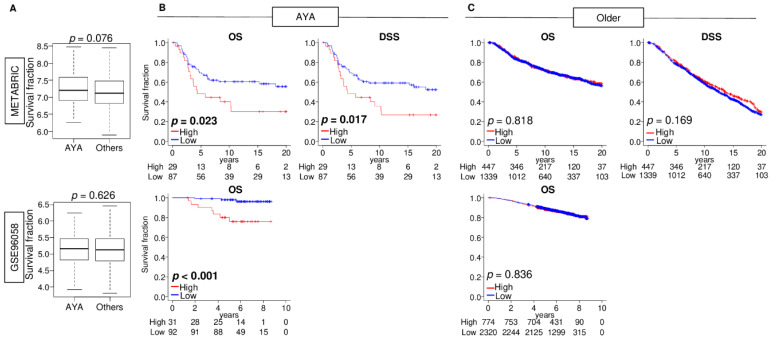
Association of *GALNT1* expression with patient survival in AYA patients and older patients. (**A**) Boxplots of *GALNT1* expression by age (AYA (less than 40 years old) vs. older (more than 40 years old)) in both METABRIC and GSE96058 cohorts. Significant difference among groups was calculated by the Mann–Whitney U tests. Kaplan–Meier plots of disease-specific survival (DSS) and overall survival (OS) in (**B**) AYA patients and (**C**) older patients in the METABRIC, and OS in GSE96058 cohorts by low (blue) and high (red) *GALNT1* expression groups. Significant difference of survival between groups was calculated by log-rank test. The top one-fourth was used as a cut-off to divide low and high groups for each cohort.

Since it is well established that breast cancer in Adolescents and Young Adults (AYA: age less than 40 years old) is typically more aggressive than in older patients, it was of interest whether *GALNT1* expression differed by age. There was no difference in *GALNT1* expression between AYA patients and older patients, which is consistent with the report on the Tn antigen [26] (Figure 2A). Interestingly, although though there was no survival difference by *GALNT1* expression in the whole breast cancer cohort, as shown in Figure 1, we found that increased *GALNT1* expression was significantly associated with worse OS and disease-specific survival (DSS) in AYA patients, whereas there was no association in the older patients in the METABRIC cohort (Figure 2B,C; *p* = 0.023 and 0.017 in AYA patients, and *p* = 0.818 and 0.169 in older patients, respectively). This association was validated in the GSE96058 cohort (*p* < 0.001). These results suggest that high levels of *GALNT1* expression is significantly associated with worse patient survival in AYA patients, but not in older patients.

### 3.3. GALNT1 Expression Was an Independent Prognostic Biomarker among AYA Patients

We next investigated the association of *GALNT1* expression with clinical features in AYA patients, but there were no associations in the two cohorts (Supplementary Figure S2). We next investigated the clinical relevance of *GALNT1* expression with clinical features in AYA patients. We found that large tumors, lymph node metastasis, and high *GALNT1* expression were all significantly associated with worse OS by univariate analysis in the AYA METABRIC cohort. Further, *GALNT1* expression and lymph node metastasis was shown to be an independent factor by multivariate analysis (Table 1; hazard ratio (HR) = 2.05 and 1.78, 95%CI 1.03–4.09 and 1.06–3.29, *p* = 0.041 and 0.029, respectively). In the AYA GSE96058 cohort, there was no statistical significance in any of the clinical features; breast cancer subtype, Nottingham histological grade, T- and N-categories of American Joint Committee on Cancer staging, whereas *GALNT1* expression was significantly associated with worse OS (Table 1; HR = 8.08, 95%CI 2.09–31.3, *p* = 0.002). These results indicated that *GALNT1* expression was an independent prognostic biomarker for breast cancer in AYA patients.

### 3.4. High GALNT1 Breast Cancer Was Significantly Associated with Decreased CD8^+^ Cell Infiltration in AYA

**Figure 3:**
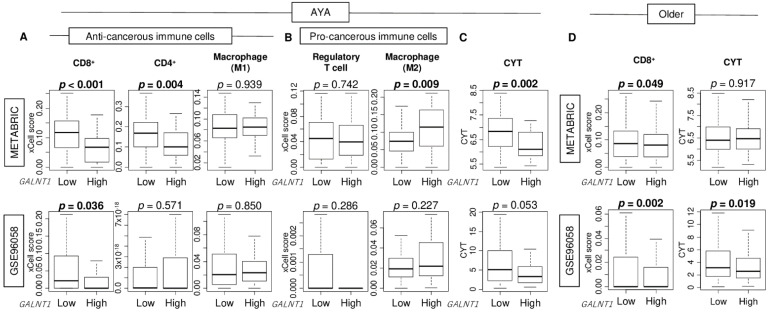
The association of the *GALNT1* expression with immunity in the tumor immune microenvironment of breast cancer. Boxplots of infiltration fraction of (**A**) anti-cancerous immune cells; CD8^+^ T cells, CD4^+^ T cells, M1 macrophages, and Th1 cells, and (**B**) pro-cancerous immune cells; regulatory T cells, M2 macrophages, and Th2 cells, and (**C**) cytolytic activity (CYT) by low and high *GALNT1* expression groups in METABRIC and GSE96058 cohorts in AYA. (**D**) CD8^+^ T cells and CYT by low and high *GALNT1* expression groups in both cohorts in older patients. Significant difference between two groups was calculated by the Mann–Whitney U test.

Given that the TN antigen has been found to suppress the tumor immune microenvironment and promote colorectal cancer growth [22], we examined the relationship between *GALNT1* expression levels and the tumor immune microenvironment in breast cancer. In AYA patients, we found that high *GALNT1* expression was significantly associated with a decreased number of CD8^+^ T cells in both the METABRIC and GSE96058 cohorts (Figure 3A; *p* < 0.001 and = 0.036, respectively). Other major immune cells, such as CD4^+^ T cells, M1 macrophages, regulatory T cells, and M2 macrophages, did not show significant difference between low and high *GALNT1* expression in the GSE96058 cohort (Figure 3A,B). Cytolytic activity, which encapsulates overall immune cell killing, was decreased in *GALNT1* high expression tumors (Figure 3C; *p* = 0.002 and = 0.053, respectively). In older breast cancer patients, high *GALNT1* expression was also significantly associated with a decreased amount of CD8^+^ T cells in both cohorts, however, the difference was smaller than that in AYA patients (Figure 3D; *p* = 0.049 and = 0.002, respectively). Further, cytolytic activity trended lower in *GALNT1* high expression tumors in older patients as well (Figure 3D; *p* = 0.917 and = 0.019, respectively). Furthermore, we investigated the relationship between *GALNT1* expression and other cells in the tumor microenvironment, including fibroblasts, microvascular and lymphatic endothelial cells, and pericytes, in AYA breast cancer. We found that only pericytes were significantly highly infiltrated in the high *GALNT1* group compared to the low group, consistently in both cohorts (Supplementary Figure S3A). There was no significant difference in mutation count between low and high *GALNT1* group in AYA patients (Supplementary Figure S3B).

### 3.5. Cancer Cell Proliferation Was Not Associated with High GALNT1 Expressed Breast Cancer in Either AYA or Older Breast Cancer Patients

**Figure 4:**
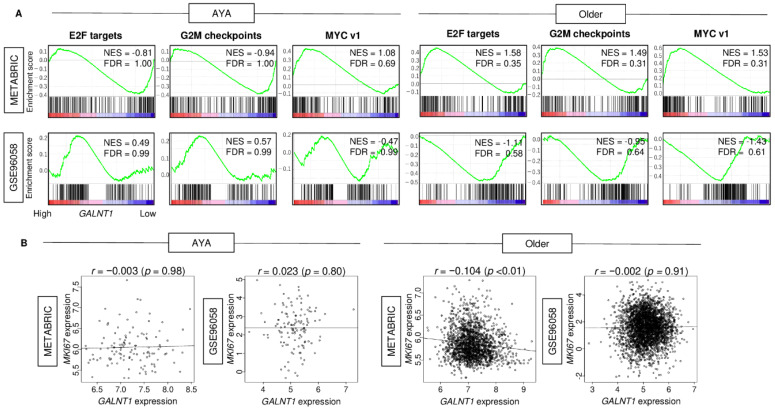
The association of high *GALNT1* breast cancer with cell proliferation signaling in AYA and older patients of the METABRIC and GSE96058 cohorts. (**A**) Enrichment plots of cell proliferation-related gene sets; E2F targets, G2M checkpoints, and MYC target v1, in high *GALNT1* expression groups of AYA and older patient in both cohorts. (**B**) Correlation plots of gene expression between *GALNT1* and *MKI67* of AYA and older patient in both cohorts. Spearman’s correlation coefficient (r) was used to the analysis. NES, normalized enrichment score.

Given that *GALNT1* expression was associated with patient survival in AYA patients but not in older patients, we hypothesized that *GALNT1* expression may have different biological roles by age group. We investigated the association between *GALNT1* expression and cell proliferation-related gene sets, which were significantly associated with breast cancer patient outcomes, as we had previously reported [43,44,45]. We found that cell proliferation-related gene sets, including E2F targets, G2M checkpoints, and MYC v1, did not demonstrate significant enrichment to high *GALNT1* expression in either AYA patients or older patients (Figure 4A). Furthermore, *GALNT1* expression was not correlated with Ki67 gene expression (*MKI67*), a cell proliferation marker used in clinics, in either age groups (Figure 4B; all Spearman’s rank correlation coefficient <0.150).

### 3.6. High GALNT1 Breast Cancer Enriched Angiogenesis, Epithelial Mesenchymal Transition (EMT), and Protein Secretion in the AYA Patient Group, but Not in the Older Patient Group

**Figure 5:**
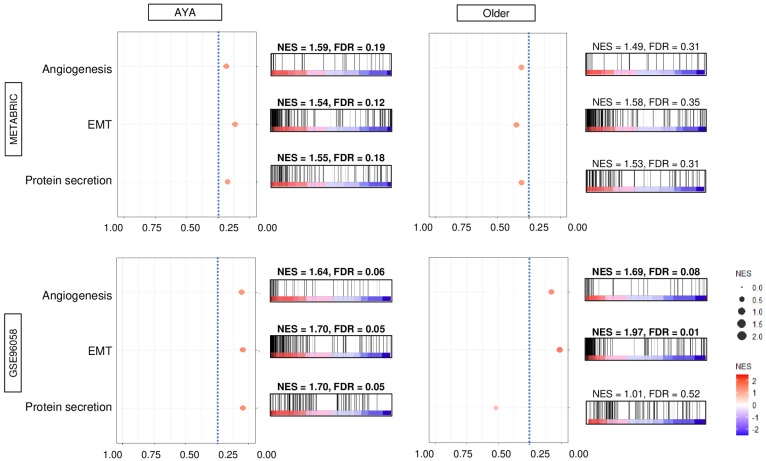
Biological feature of high *GALNT1* breast cancer in AYA patients and older patients of the METABRIC and GSE96058 cohorts. Dot plots with distribution map of the three gene sets, which showed significant differences consistently in both cohorts, with the lowest FDR q-value in the enrichment analysis between *GALNT1* low- and high-expression breast cancer groups using the GSEA algorithm. The top one-fourth was used as a cut-off to divide low and high groups. FDR, false discovery rate; NES, normalized enrichment score.

Finally, we investigated the biological functions using a hallmark gene set enrichment analysis. We found that breast cancer with high *GALNT1* expression in AYA patients significantly enriched angiogenesis, EMT, and protein secretion gene sets, consistently in both the METABRIC and GSE96058 cohorts (Figure 5). In older patients; however, there were no hallmark gene sets that were significantly enriched to high *GALNT1* expression consistently in both cohorts.

## 4. Discussion

*GALNT1* expression, which generates the Tn antigen, was associated with decreased infiltration of CD8 T cells, enhanced angiogenesis, EMT, and protein secretion, and was a prognostic biomarker for breast cancer in AYA patients, but not older patients. Amongst both breast cancer cohorts, *GALNT1* expression level was highest in TNBC, but was not associated with patient survival or clinical aggressiveness, including histological grade and metastasis. None of the hallmark cancer pathways, except for KRAS, were significantly enriched to high *GALNT1* expression breast cancer by GSEA. Although there was no significant difference in *GALNT1* expression levels between AYA patients and older patients, high *GALNT1* expression was associated with significantly worse patient survival in AYA patients but not in older patients consistently in two independent cohorts. We found that high *GALNT1* expression was an independent prognostic factor among several clinical features, including subtype, histological grade, AJCC T and N-category, in the AYA generation of both cohorts. Further, breast cancer with high *GALNT1* expression was associated with less infiltration of CD8^+^ T cells, but not other immune cells. Increased levels of *GALNT1* expression in breast cancer amongst AYA patients enriched angiogenesis, EMT, and protein secretion but not in older patients.

Altered levels of *GALNT* expression or distribution have been extensively reported in a wide range of cancer types [14]. *GALNT* expression results in marked alterations in GalNAc O-linked glycosylation, including exposure of truncated O-glycans such as the Tn antigen [13,46]. Altered levels of specific GALNTs were shown to have prognostic significance in cancers and were associated with changes in cell behavior, including cell proliferation, migration, invasion and metastasis in animal models [47]. In breast cancer, *GALNT4* [48], *GALNT*6 [49], and *GALNT*8 [50], have been reported to be associated with patient outcomes, whereas no other study examines the clinical role of *GALNT1*. *GALNT1* initiates O-glycosylation of glycoproteins; Sonic hedgehog protein (Shh), osteopontin, and MUC1, which contributes to the generation of breast cancer [14]. Although the role of *GALNT1* in breast carcinogenesis is known, the clinical significance of its expression in breast cancer progression remains unclear. To our knowledge, our study is the first to report that *GALNT1* mRNA expression levels are associated with decreased CD8 T-cell infiltration, angiogenesis and EMT, and poorer survival outcomes in AYA patients.

The Tn antigen is generated by *GALNT* and is present in 50–90% of all types of cancer, where it is associated with metastasis and decreased survival. Breast cancer was reported to have high levels of the Tn antigen [51]. Maria Florencia Festari et al. reported that the Tn antigen enhances breast tumor growth and lung metastasis by immunosuppression and suggested the Tn antigen-based immunotherapy approach [52]. Our study showed no significant association of *GALNT1* expression with cell proliferation or metastasis. Further, *GALNT1* expression was not associated with high infiltration of pro-cancerous immune cells, but with low infiltration of CD8^+^ T cells, which was evident in both the AYA generation and older patients, with the difference more pronounced in the AYA generation.

Data remains controversial regarding biological characteristics amongst breast tumors in AYA patients and older women. Although there have been several studies on genes that are differentially expressed between AYA patients and older patients, *GALNT1*, which has clinical significance in the AYA generation—with no difference in gene expression levels between generations—is unique. As the number of drug therapies for breast cancer has been rapidly expanding in recent years, the association between *GALNT1* expression and response for other drugs warrants further exploration, particularly for drugs that target angiogenesis or EMT. For instance, the anti-angiogenesis approach clinical trial which failed for breast cancer may turn out to be effective for AYA patients. We cannot help but speculate that our findings on *GALNT1* expression demonstrate its potential to become a predictive biomarker and a novel therapeutic target for breast cancer in the AYA generation [53].

Although intriguing, the data presented is still preliminary and requires additional validation through future research. Once validated, this information may prove beneficial to AYA breast cancer patients, and their physicians. As genotyping becomes increasingly more common in clinical decision making, this information may be especially vital in treatment decision making for early stage and/or advanced breast cancer. Remaining unanswered questions include how *GALNT1* interacts with various pathways and immune cells.

## 5. Conclusions

*GALNT1* expression was significantly associated with worse survival and associated with cell proliferation signaling in the AYA generation but not in older patients. *GALNT1* expression may have potential as a predictive biomarker amongst AYA patients with breast cancer.

## Figures and Tables

**Table 1:** Uni- and multivariate analysis in AYA of the METABRIC and GSE96058 cohorts.

METABRIC (OS)		Univariate				Multivariate				GSE96058 (OS)		Univariate			
		HR	95%CI	*p*		HR	95%CI	*p*				HR	95%CI	*p*	
Subtype	TNBC vs. others	1.11	0.65–1.89	0.708						Subtype	TNBC vs. others	1.35	0.15–12.1	0.790	
Grade	G3 vs. G1/2	1.73	0.85–3.53	0.134						Grade	G3 vs. G1/2	2.19	0.46–10.3	0.322	
T	T3/4 vs. T1/2	0.52	0.29–0.92	0.026	*	0.69	0.37–1.27	0.23		T	T3/4 vs. T1/2	-	-		
N	N+ vs. N−	2.63	1.39–4.99	0.003	*	2.05	1.03–4.09	0.041	*	N	N+ vs. N−	0.78	0.16–3.73	0.751	
*GALNT1*	High vs. Low	2.36	1.32–4.20	0.004	*	1.87	1.06–3.29	0.029	*	*GALNT1*	High vs. Low	8.08	2.09–31.3	0.002	*

*; significant difference.

## Data Availability

All the cohorts/datasets used in this study; Molecular Taxonomy of Breast Cancer International Consortium (METABRIC) and GSE96058 are all publicly available without any restrictions via cBioportal or Gene Expression Omnibus (GEO).

## Supplementary Materials

Supplementary materials can be found at https://www.mdpi.com/article/10.3390/cancers15133489/s1. Supplementary Figure S1: Association of *GALNT1* expression with clinicopathlogical features in the GSE96058 cohort. Figure S2: Association of *GALNT1* expression with clinical aggressiveness in AYA patients. Figure S3: Association of *GALNT1* expression with stromal fraction and mutation count in breast cancer of AYA patients.

## Abbreviations

AJCC	American Joint Committee on Cancer
DFS	disease-free survival
DSS	disease-specific survival
ER	estrogen receptor
FDR	false discovery rate
GSEA	gene set enrichment analysis
HER2	human epidermal growth factor receptor 2
METABRIC	Molecular Taxonomy of Breast Cancer International Consortium
NES	normalized enrichment score
OS	overall survival
TNBC	triple-negative breast cancer
