# Supplementary material for: GALNT1 Expression Is Associated with Angiogenesis and Is a Prognostic Biomarker for Breast Cancer in Adolescents and Young Adults (AYA)
Source: Cancers (Basel). 2023 Jul 4;15(13):3489. doi: 10.3390/cancers15133489 (PMC10341315; doi:10.3390/cancers15133489)
Supplement: Supplementary file 1 [file cancers-15-03489-s001.zip › cancers-2456564-supplementary.pdf]

SUPPLEMENTAL MATERIALS

Oshi et al. "*GALNT1* expression is associated with angiogenesis and is a prognostic biomarker for breast cancer in adolescents and young adults (AYA)"

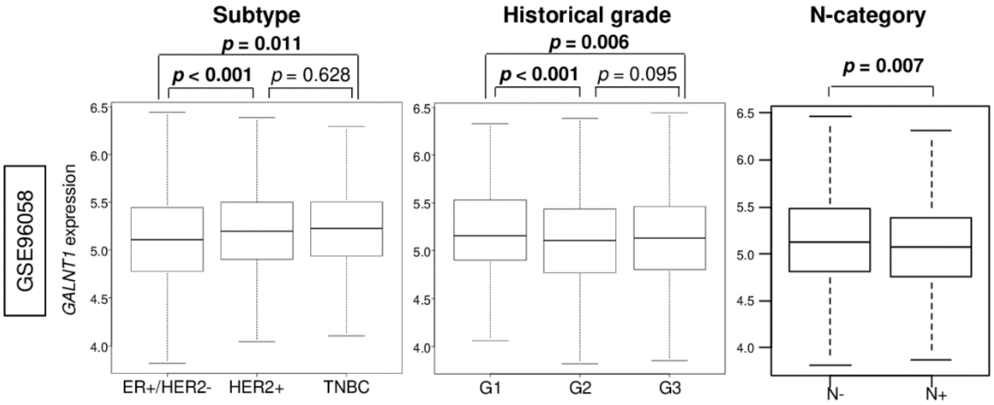

Figure S1. Association of *GALNT1* expression with clinicopathological features in the GSE96058 cohort. Boxplots of *GALNT1* expression by subtype (ER-positive/HER2-negative, HER2-positive, and triple-negative breast cancer (TNBC)), histological grade (G1-G3), and lymph node metastasis (N-negative and N-positive) in the GSE96058 cohort. Significant difference among groups was calculated by the Kruskal-Wallis and Mann-Whitney U tests.

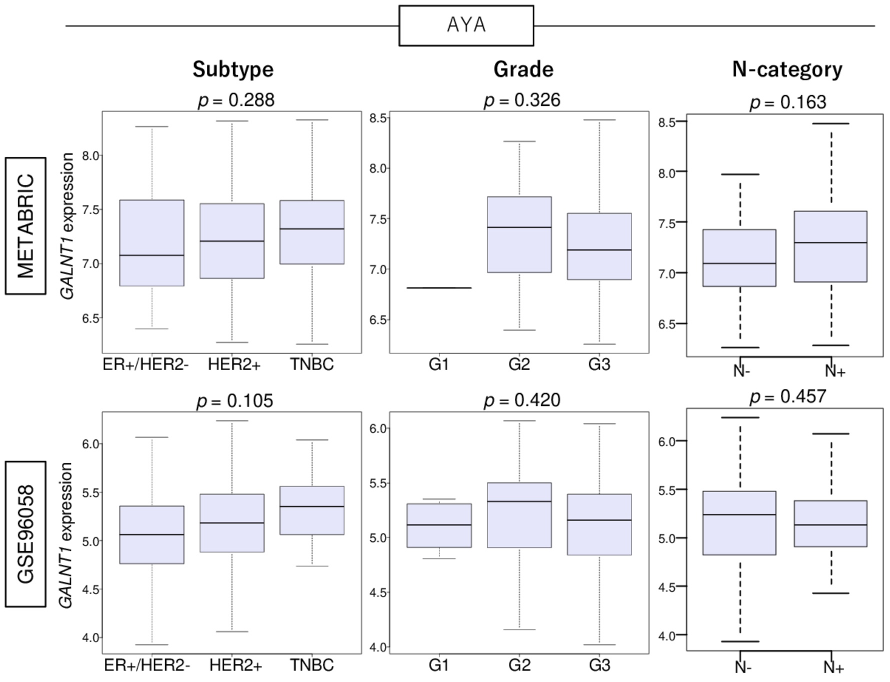

Figure S2. Association of *GALNT1* expression with clinical aggressiveness in AYA patients. Boxplots of *GALNT1* expression by clinical factors; subtypes (ER+/HER2-, HER2+, TNBC), Nottingham grade (G1-3), and lymph node metastasis (N-/N+), in the METABRIC and GSE96058 cohorts. Significant difference between groups was calculated by the Kruskal-Wallis and Mann-Whitney U test. ER, estrogen receptor; HER, human epidermal growth factor receptor; TNBC, triple-negative breast cancer.

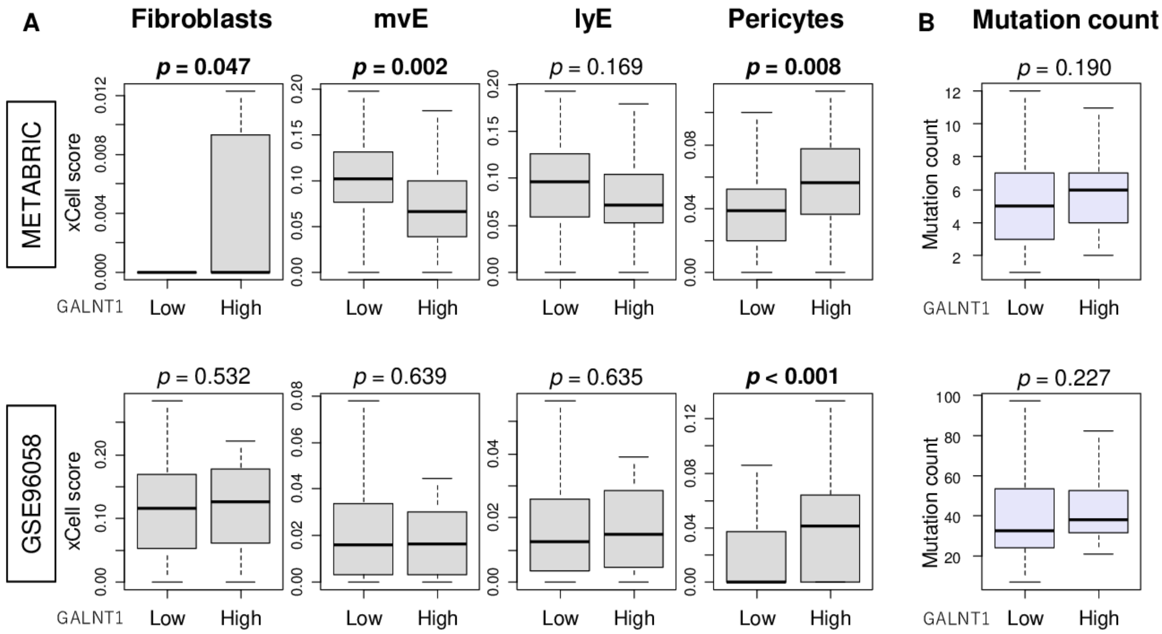

Figure S3. Association of *GALNT1* expression with stromal fraction and mutation count in breast cancer of AYA patients. Boxplots of the infiltration fraction of stromal cells; fibroblasts, mvE (microvascular endothelial cells), lyE (lymphatic endothelial cells), and pericytes by low and high *GALNT1* expression groups in the METABRIC and GSE96058 cohorts. **(B)** Boxplots of mutation count by low and high *GALNT1* expression groups in both cohorts. Significant difference between two groups was calculated by the Mann-Whitney U test.
